# (Dithio­benzoato-κ^2^ *S*,*S*′)[hydridotris(pyrazol-1-yl-κ*N* ^2^)borato](triphenyl­phosphine-κ*P*)ruthenium(II)

**DOI:** 10.1107/S1600536808036702

**Published:** 2008-11-13

**Authors:** Chia-Her Lin, Yao-Ren Liang, Hung-Chun Tong, Yih Hsing Lo, Ting Shen Kuo

**Affiliations:** a Department of Chemistry, Chung-Yuan Christian University, Chung-Li 320, Taiwan; b Department of Chemical Engineering, Tatung University, Taipei 104, Taiwan; c Department of Chemistry, National Normal Taiwan University, Taipei 106, Taiwan

## Abstract

Reaction of [Ru(Tp)Cl(PPh_3_)_2_] (Tp = hydridotrispyrazolyl­borate) with ammonium dithio­benzoate in methanol leads to the formation of the title compound, [Ru(C_9_H_10_BN_6_)(C_7_H_5_S_2_)(C_18_H_15_P)]. In the crystal structure, the Ru atom is coordinated by three N atoms of the Tp ligand, one P atom of the triphenyl­phosphine ligand and the two S atoms of the dithio­benzoate ligand within a slightly distorted octa­hedron. The Ru—S bonds are slightly different [2.321 (1) and 2.396 (1) Å] and the average N—Ru—N angle is 86.31°.

## Related literature

For general background, see: Alock *et al.* (1992 ▶); Burrows (2001 ▶); Pavlik *et al.* (2005 ▶); Hidai *et al.* (2000 ▶); Vit & Zdrazil (1989 ▶). For related structures, see: Gemel *et al.* (1996 ▶); Slugovc *et al.* (1998 ▶); Sellmann *et al.* (1999 ▶); Meno *et al.* (1995 ▶).
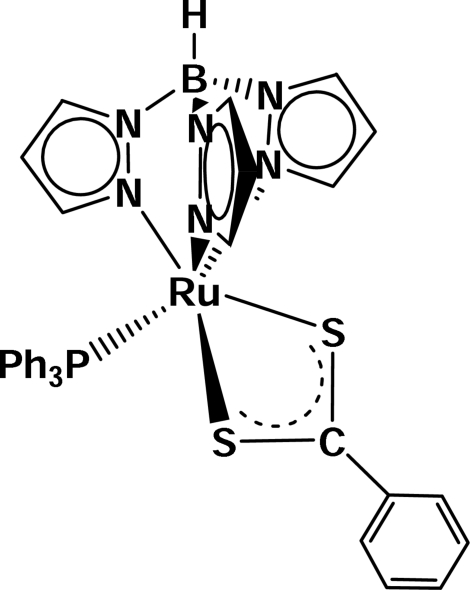


## Experimental

### 

#### Crystal data

[Ru(C_9_H_10_BN_6_)(C_7_H_5_S_2_)(C_18_H_15_P)]
*M* *_r_* = 729.61
Monoclinic, 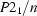

*a* = 12.8915 (13) Å
*b* = 18.394 (2) Å
*c* = 13.5174 (16) Å
β = 96.591 (5)°
*V* = 3184.2 (6) Å^3^
*Z* = 4
Mo *K*α radiation
μ = 0.71 mm^−1^
*T* = 200 (2) K
0.18 × 0.12 × 0.02 mm

#### Data collection

Nonius KappaCCD diffractometer
Absorption correction: multi-scan (Blessing, 1995 ▶) *T*_min_ = 0.883, *T*_max_ = 0.986
22786 measured reflections
5557 independent reflections
3974 reflections with *I* > 2σ(*I*)
*R*_int_ = 0.056

#### Refinement

*R*[*F* ^2^ > 2σ(*F* ^2^)] = 0.036
*wR*(*F* ^2^) = 0.080
*S* = 1.02
5557 reflections
406 parameters
H-atom parameters constrained
Δρ_max_ = 1.39 e Å^−3^
Δρ_min_ = −0.62 e Å^−3^

### 

Data collection: *COLLECT* (Nonius, 1999 ▶); cell refinement: *DENZO* and *SCALEPACK* (Otwinowski & Minor, 1997 ▶); data reduction: *DENZO* and *SCALEPACK*; program(s) used to solve structure: *SHELXS97* (Sheldrick, 2008 ▶); program(s) used to refine structure: *SHELXL97* (Sheldrick, 2008 ▶); molecular graphics: *ORTEP-3 for Windows* (Farrugia, 1997 ▶); software used to prepare material for publication: *WinGX* (Farrugia, 1999 ▶).

## Supplementary Material

Crystal structure: contains datablocks I, global. DOI: 10.1107/S1600536808036702/nc2121sup1.cif

Structure factors: contains datablocks I. DOI: 10.1107/S1600536808036702/nc2121Isup2.hkl

Additional supplementary materials:  crystallographic information; 3D view; checkCIF report

## Figures and Tables

**Fig. 1.**
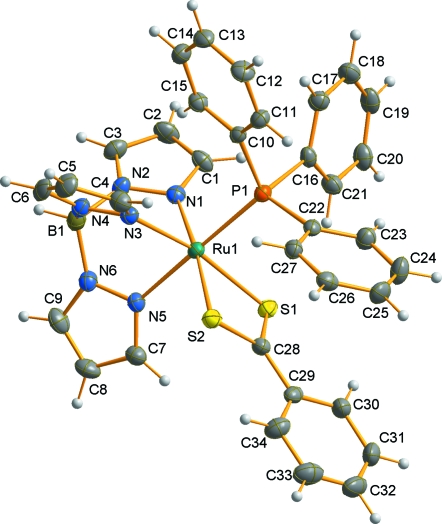
Molecular structure of the title compound with labelling and displacement ellipsoids drawn at the 30% probability level (H atoms are shown as spheres of arbitrary radius).

## supplementary crystallographic information

### Comment

Ruthenium(II) hydridotripyrazolylborate complexes, (Ru(Tp), are of interest for stoichiometric and catalytic transformations of organic molecules (Pavlik *et al.*, 2005). The complex [Ru(Tp)Cl(PPh_3_)_2_] (Alock *et al.*, 1992) has been used as the starting material for the synthesis of several complexes because the chloride atom and the phosphine ligand can easily be substituted (Burrows, 2001). On the other hand, the chemistry of transition metal sulfur compounds has attracted much interest for their importance in the field of catalysis and metalloenzymes (Hidai *et al.*, 2000). In recent years there has been an increased interest in ruthenium sulfur complexes, in part because of the high catalytic activity of RuS_2_ in various hydrotreating processes (Vit & Zdrazil, 1989). Thus, many ruthenium thiolate complexes have been reported. However, ruthenium complexes with dithio ligands are relatively rare (Sellmann *et al.*, 1999). In this context the structure of the title compound was determined.

In the crystal structure of the title compound, the Ru atom is coordinated by three N atoms of the Tp ligand, two S atoms of the dithiobenzoate ligand and one P atom of the triphenylphosphine ligand forming slightly distorted octahedron. The  average N—Ru—N angle amount to 86.31° and the three Ru—N bond lengths of 2.141 (3), 2.098 (3) and 2.134 (3) Å are slightly longer than the average distance of 2.038 Å in observed in other RuTp complexes (Gemel *et al.*1996 and Slugovc *et al.*1998). The dithiobenzoate ligand chelates the ruthenium centre with slightly different Ru—S bonds of 2.321 (1) Å and and 2.396 (1) Å and an S—Ru—S angle of 71.61 (3)°. The average Ru—S bond length of 2.3588 (11) Å is slightly shorter than in *cis*-[Ru(S_2_CNEt_2_)_2_(PPh_3_)_2_] (av. 2.3952 (5) Å) (Meno *et al.*, 1995).

### Experimental

To a solution of [Ru(Tp)Cl(PPh_3_)_2_] (3.95 g, 4.50 mmol) in MeOH (20 ml) an excess of [NH_4_][S_2_C(C_6_H_5_)] (1.71 g, 10 mmol) were added. The reaction mixture was stirred for 4 h at room temperature. The solvent was removed in vacuum and 20 ml of CH_2_Cl_2_ were added to the residue. After filtration the solvent was removed in vacuum to give the title compound. Spectroscopic analysis: IR(KBr, cm^-1^): ν(BH)2467 cm^-1^.^31^P NMR(CDCl_3_, 303 K, δ,p.p.m.): d 58.3 (PPh_3_). MS (*m*/*z*,Ru102): 730.2 (*M*^+^), 468.1(*M*^+^ - PPh_3_). Anal. Calcd for C_34_H_30_BN_6_PRuS_2_: C, 55.97; H,4.14; N, 11.52. Found: C, 55.73; H, 4.11;N, 11.42. The bright-yellow crystals used for X-ray structure analysis were obtained by recrystallization of the crude product from dichloromethane–hexane.

### Refinement

The H atoms were placed in idealized positions and constrained to ride on their parent atoms, with C—H = 0.95 Å and *U*_iso_(H) = 1.2*U*_eq_(C), B—H = 1.0 Å and *U*_iso_(H) = 1.2*U*_eq_(B).

### Crystal data

**Table:**

[Ru(C_9_H_10_BN_6_)(C_7_H_5_S_2_)(C_18_H_15_P)]	*F*_000_ = 1488
*M**_r_* = 729.61	*D*_x_ = 1.522 Mg m^−^^3^
Monoclinic, *P*2_1_/*n*	Mo *K*α radiation λ = 0.71073 Å
Hall symbol: -P 2yn	Cell parameters from 5326 reflections
*a* = 12.8915 (13) Å	θ = 2.3–24.9º
*b* = 18.394 (2) Å	µ = 0.71 mm^−^^1^
*c* = 13.5174 (16) Å	*T* = 200 (2) K
β = 96.591 (5)º	Prism, green
*V* = 3184.2 (6) Å^3^	0.18 × 0.12 × 0.02 mm
*Z* = 4	

### Data collection

**Table:**

Nonius KappaCCD diffractometer	5557 independent reflections
Radiation source: fine-focus sealed tube	3974 reflections with *I* > 2σ(*I*)
Monochromator: graphite	*R*_int_ = 0.056
*T* = 200(2) K	θ_max_ = 25.0º
CCD rotation images, thick slices scans	θ_min_ = 1.9º
Absorption correction: multi-scan(Blessing, 1995)	*h* = −15→12
*T*_min_ = 0.883, *T*_max_ = 0.986	*k* = −21→20
22786 measured reflections	*l* = −16→15

### Refinement

**Table:**

Refinement on *F*^2^	Secondary atom site location: difference Fourier map
Least-squares matrix: full	Hydrogen site location: inferred from neighbouring sites
*R*[*F*^2^ > 2σ(*F*^2^)] = 0.036	H-atom parameters constrained
*wR*(*F*^2^) = 0.080	*w* = 1/[σ^2^(*F*_o_^2^) + (0.0315*P*)^2^ + 1.684*P*] where *P* = (*F*_o_^2^ + 2*F*_c_^2^)/3
*S* = 1.02	(Δ/σ)_max_ = 0.001
5557 reflections	Δρ_max_ = 1.39 e Å^−^^3^
406 parameters	Δρ_min_ = −0.62 e Å^−^^3^
Primary atom site location: structure-invariant direct methods	Extinction correction: none

### Special details

**Table:**

Experimental. Semi-empirical from equivalents by WinGX (Blessing, 1995)
Geometry. All e.s.d.'s (except the e.s.d. in the dihedral angle between two l.s. planes) are estimated using the full covariance matrix. The cell e.s.d.'s are taken into account individually in the estimation of e.s.d.'s in distances, angles and torsion angles; correlations between e.s.d.'s in cell parameters are only used when they are defined by crystal symmetry. An approximate (isotropic) treatment of cell e.s.d.'s is used for estimating e.s.d.'s involving l.s. planes.
Refinement. Refinement of *F*^2^ against ALL reflections. The weighted *R*-factor *wR* and goodness of fit *S* are based on *F*^2^, conventional *R*-factors *R* are based on *F*, with *F* set to zero for negative *F*^2^. The threshold expression of *F*^2^ > σ(*F*^2^) is used only for calculating *R*-factors(gt) *etc*. and is not relevant to the choice of reflections for refinement. *R*-factors based on *F*^2^ are statistically about twice as large as those based on *F*, and *R*- factors based on ALL data will be even larger.

### Fractional atomic coordinates and isotropic or equivalent isotropic displacement parameters (Å^2^)

**Table:**

	*x*	*y*	*z*	*U*_iso_*/*U*_eq_	
B1	0.3330 (3)	0.7338 (2)	0.0521 (3)	0.0277 (11)	
H1'	0.3875	0.7068	0.0198	0.033*	
C1	0.3375 (3)	0.75212 (19)	0.3191 (3)	0.0272 (9)	
H1	0.3096	0.7678	0.3777	0.033*	
C2	0.4285 (3)	0.7130 (2)	0.3191 (3)	0.0331 (10)	
H2	0.4739	0.6970	0.3752	0.040*	
C3	0.4393 (3)	0.7023 (2)	0.2210 (3)	0.0283 (9)	
H3	0.4949	0.6770	0.1959	0.034*	
C4	0.2720 (3)	0.9240 (2)	0.0120 (3)	0.0282 (9)	
H4	0.2359	0.9673	0.0255	0.034*	
C5	0.3432 (3)	0.9186 (2)	−0.0566 (3)	0.0334 (10)	
H5	0.3648	0.9559	−0.0983	0.040*	
C6	0.3760 (3)	0.8478 (2)	−0.0514 (3)	0.0310 (10)	
H6	0.4256	0.8267	−0.0897	0.037*	
C7	0.0588 (3)	0.68809 (19)	0.0294 (3)	0.0259 (9)	
H7	−0.0109	0.6970	0.0424	0.031*	
C8	0.0901 (3)	0.6302 (2)	−0.0258 (3)	0.0338 (10)	
H8	0.0474	0.5927	−0.0570	0.041*	
C9	0.1945 (3)	0.6385 (2)	−0.0257 (3)	0.0327 (10)	
H9	0.2389	0.6070	−0.0574	0.039*	
C10	0.2846 (3)	0.98437 (19)	0.2445 (2)	0.0191 (8)	
C11	0.2725 (3)	1.05912 (19)	0.2465 (3)	0.0243 (9)	
H11	0.2057	1.0794	0.2525	0.029*	
C12	0.3569 (3)	1.1045 (2)	0.2400 (3)	0.0298 (10)	
H12	0.3478	1.1557	0.2408	0.036*	
C13	0.4542 (3)	1.0757 (2)	0.2322 (3)	0.0279 (9)	
H13	0.5124	1.1070	0.2287	0.034*	
C14	0.4670 (3)	1.0014 (2)	0.2297 (3)	0.0280 (9)	
H14	0.5341	0.9814	0.2242	0.034*	
C15	0.3825 (3)	0.9555 (2)	0.2350 (3)	0.0241 (9)	
H15	0.3916	0.9043	0.2323	0.029*	
C16	0.2187 (3)	0.89959 (19)	0.3980 (3)	0.0218 (8)	
C17	0.2997 (3)	0.9362 (2)	0.4538 (3)	0.0275 (9)	
H17	0.3328	0.9756	0.4247	0.033*	
C18	0.3329 (3)	0.9164 (2)	0.5511 (3)	0.0349 (10)	
H18	0.3890	0.9418	0.5877	0.042*	
C19	0.2853 (3)	0.8606 (2)	0.5942 (3)	0.0329 (10)	
H19	0.3102	0.8460	0.6601	0.040*	
C20	0.2015 (3)	0.8251 (2)	0.5433 (3)	0.0322 (10)	
H20	0.1661	0.7878	0.5747	0.039*	
C21	0.1693 (3)	0.8448 (2)	0.4452 (3)	0.0273 (9)	
H21	0.1120	0.8200	0.4096	0.033*	
C22	0.0647 (3)	0.97588 (18)	0.2688 (3)	0.0206 (8)	
C23	0.0053 (3)	0.9746 (2)	0.3480 (3)	0.0301 (10)	
H23	0.0297	0.9485	0.4068	0.036*	
C24	−0.0893 (3)	1.0110 (2)	0.3421 (3)	0.0380 (11)	
H24	−0.1290	1.0100	0.3971	0.046*	
C25	−0.1262 (3)	1.0486 (2)	0.2574 (3)	0.0341 (10)	
H25	−0.1921	1.0722	0.2530	0.041*	
C26	−0.0671 (3)	1.0517 (2)	0.1791 (3)	0.0279 (9)	
H26	−0.0913	1.0787	0.1212	0.034*	
C27	0.0272 (3)	1.01580 (19)	0.1842 (3)	0.0228 (9)	
H27	0.0673	1.0182	0.1295	0.027*	
C28	−0.0643 (3)	0.83083 (19)	0.1494 (3)	0.0212 (8)	
C29	−0.1777 (3)	0.83849 (19)	0.1434 (3)	0.0256 (9)	
C30	−0.2313 (3)	0.8185 (2)	0.2228 (3)	0.0341 (10)	
H30	−0.1940	0.8001	0.2822	0.041*	
C31	−0.3392 (3)	0.8253 (3)	0.2156 (3)	0.0454 (12)	
H31	−0.3755	0.8108	0.2697	0.055*	
C32	−0.3936 (3)	0.8529 (2)	0.1303 (4)	0.0457 (12)	
H32	−0.4673	0.8575	0.1258	0.055*	
C33	−0.3420 (3)	0.8735 (2)	0.0523 (4)	0.0452 (12)	
H33	−0.3801	0.8927	−0.0063	0.054*	
C34	−0.2346 (3)	0.8668 (2)	0.0578 (3)	0.0334 (10)	
H34	−0.1993	0.8815	0.0032	0.040*	
N1	0.2933 (2)	0.76531 (15)	0.2267 (2)	0.0215 (7)	
N2	0.3582 (2)	0.73352 (16)	0.1657 (2)	0.0235 (7)	
N3	0.2606 (2)	0.86080 (16)	0.0565 (2)	0.0210 (7)	
N4	0.3261 (2)	0.81318 (16)	0.0171 (2)	0.0241 (7)	
N5	0.1408 (2)	0.72945 (15)	0.0615 (2)	0.0223 (7)	
N6	0.2250 (2)	0.69857 (16)	0.0266 (2)	0.0251 (7)	
P1	0.18113 (7)	0.91954 (5)	0.26504 (7)	0.0190 (2)	
Ru1	0.16002 (2)	0.822395 (15)	0.15631 (2)	0.01805 (9)	
S1	0.01327 (7)	0.78411 (5)	0.23709 (7)	0.0227 (2)	
S2	0.00968 (7)	0.87056 (5)	0.06980 (7)	0.0231 (2)	

### Atomic displacement parameters (Å^2^)

**Table:**

	*U*^11^	*U*^22^	*U*^33^	*U*^12^	*U*^13^	*U*^23^
B1	0.025 (3)	0.031 (3)	0.028 (3)	0.005 (2)	0.006 (2)	−0.001 (2)
C1	0.038 (3)	0.022 (2)	0.020 (2)	0.0017 (18)	−0.0020 (18)	0.0005 (17)
C2	0.036 (3)	0.033 (2)	0.028 (2)	0.0073 (19)	−0.0084 (19)	0.0016 (19)
C3	0.021 (2)	0.026 (2)	0.037 (3)	0.0045 (17)	0.0015 (18)	0.0056 (19)
C4	0.033 (2)	0.026 (2)	0.024 (2)	−0.0042 (18)	0.0010 (18)	0.0035 (18)
C5	0.034 (3)	0.036 (3)	0.032 (2)	−0.0045 (19)	0.0105 (19)	0.009 (2)
C6	0.025 (2)	0.047 (3)	0.023 (2)	−0.0026 (19)	0.0100 (17)	0.0058 (19)
C7	0.028 (2)	0.025 (2)	0.024 (2)	−0.0035 (18)	0.0006 (17)	0.0000 (17)
C8	0.037 (3)	0.029 (2)	0.033 (3)	−0.0087 (19)	−0.0037 (19)	−0.004 (2)
C9	0.048 (3)	0.022 (2)	0.029 (2)	0.0042 (19)	0.004 (2)	−0.0041 (19)
C10	0.017 (2)	0.023 (2)	0.018 (2)	−0.0013 (16)	0.0032 (15)	0.0009 (16)
C11	0.020 (2)	0.025 (2)	0.028 (2)	0.0013 (16)	0.0048 (17)	0.0001 (18)
C12	0.035 (3)	0.019 (2)	0.036 (2)	−0.0033 (18)	0.0028 (19)	−0.0010 (18)
C13	0.027 (2)	0.030 (2)	0.027 (2)	−0.0109 (18)	0.0061 (17)	−0.0006 (18)
C14	0.021 (2)	0.032 (2)	0.032 (2)	0.0007 (17)	0.0050 (17)	−0.0034 (19)
C15	0.027 (2)	0.020 (2)	0.025 (2)	−0.0008 (17)	0.0032 (17)	−0.0006 (17)
C16	0.024 (2)	0.024 (2)	0.018 (2)	0.0070 (16)	0.0031 (16)	−0.0004 (16)
C17	0.025 (2)	0.031 (2)	0.026 (2)	0.0006 (17)	0.0016 (17)	0.0015 (19)
C18	0.029 (2)	0.046 (3)	0.028 (2)	0.004 (2)	−0.0019 (18)	−0.005 (2)
C19	0.035 (3)	0.043 (3)	0.020 (2)	0.015 (2)	0.0022 (19)	0.004 (2)
C20	0.041 (3)	0.032 (2)	0.024 (2)	0.007 (2)	0.0088 (19)	0.005 (2)
C21	0.031 (2)	0.028 (2)	0.022 (2)	−0.0023 (17)	0.0015 (17)	0.0000 (17)
C22	0.020 (2)	0.0188 (19)	0.023 (2)	−0.0035 (16)	0.0047 (16)	−0.0039 (17)
C23	0.027 (2)	0.035 (2)	0.029 (2)	0.0039 (19)	0.0049 (18)	0.0014 (19)
C24	0.035 (3)	0.046 (3)	0.035 (3)	0.009 (2)	0.016 (2)	0.001 (2)
C25	0.021 (2)	0.040 (3)	0.042 (3)	0.0084 (18)	0.004 (2)	−0.006 (2)
C26	0.027 (2)	0.026 (2)	0.030 (2)	0.0022 (17)	−0.0036 (18)	−0.0047 (18)
C27	0.023 (2)	0.024 (2)	0.023 (2)	−0.0002 (16)	0.0053 (16)	−0.0001 (17)
C28	0.021 (2)	0.023 (2)	0.020 (2)	−0.0022 (16)	0.0010 (15)	−0.0024 (17)
C29	0.019 (2)	0.023 (2)	0.034 (2)	−0.0022 (16)	0.0034 (18)	−0.0101 (18)
C30	0.025 (2)	0.050 (3)	0.028 (2)	−0.003 (2)	0.0047 (18)	−0.013 (2)
C31	0.030 (3)	0.068 (3)	0.041 (3)	−0.004 (2)	0.015 (2)	−0.025 (3)
C32	0.022 (3)	0.053 (3)	0.062 (3)	0.004 (2)	0.002 (2)	−0.023 (3)
C33	0.031 (3)	0.043 (3)	0.059 (3)	0.004 (2)	−0.007 (2)	−0.004 (2)
C34	0.025 (3)	0.032 (2)	0.043 (3)	0.0008 (18)	0.0023 (19)	0.005 (2)
N1	0.0232 (18)	0.0173 (16)	0.0244 (18)	−0.0015 (13)	0.0042 (14)	0.0000 (14)
N2	0.0193 (18)	0.0260 (17)	0.0255 (18)	0.0024 (14)	0.0047 (14)	0.0024 (15)
N3	0.0207 (18)	0.0228 (17)	0.0195 (17)	−0.0011 (13)	0.0020 (13)	0.0022 (14)
N4	0.0198 (17)	0.0311 (19)	0.0218 (17)	0.0011 (14)	0.0043 (13)	0.0026 (15)
N5	0.0238 (19)	0.0212 (17)	0.0221 (17)	0.0006 (14)	0.0032 (14)	−0.0026 (14)
N6	0.0270 (19)	0.0232 (18)	0.0257 (19)	0.0004 (14)	0.0067 (14)	−0.0014 (14)
P1	0.0186 (5)	0.0182 (5)	0.0203 (5)	−0.0001 (4)	0.0030 (4)	0.0008 (4)
Ru1	0.01702 (17)	0.01847 (15)	0.01893 (17)	−0.00125 (13)	0.00328 (11)	0.00074 (14)
S1	0.0213 (5)	0.0233 (5)	0.0239 (5)	−0.0014 (4)	0.0042 (4)	0.0021 (4)
S2	0.0216 (6)	0.0252 (5)	0.0225 (5)	−0.0010 (4)	0.0016 (4)	0.0013 (4)

### Geometric parameters (Å, °)

**Table:**

B1—N2	1.533 (5)	C18—C19	1.361 (5)
B1—N4	1.535 (5)	C18—H18	0.9500
B1—N6	1.538 (5)	C19—C20	1.378 (5)
B1—H1'	1.0000	C19—H19	0.9500
C1—N1	1.335 (4)	C20—C21	1.391 (5)
C1—C2	1.376 (5)	C20—H20	0.9500
C1—H1	0.9500	C21—H21	0.9500
C2—C3	1.363 (5)	C22—C23	1.386 (5)
C2—H2	0.9500	C22—C27	1.398 (5)
C3—N2	1.342 (4)	C22—P1	1.829 (4)
C3—H3	0.9500	C23—C24	1.386 (5)
C4—N3	1.325 (4)	C23—H23	0.9500
C4—C5	1.382 (5)	C24—C25	1.375 (5)
C4—H4	0.9500	C24—H24	0.9500
C5—C6	1.368 (5)	C25—C26	1.375 (5)
C5—H5	0.9500	C25—H25	0.9500
C6—N4	1.347 (4)	C26—C27	1.378 (5)
C6—H6	0.9500	C26—H26	0.9500
C7—N5	1.334 (4)	C27—H27	0.9500
C7—C8	1.386 (5)	C28—C29	1.461 (5)
C7—H7	0.9500	C28—S2	1.684 (4)
C8—C9	1.355 (5)	C28—S1	1.694 (4)
C8—H8	0.9500	C29—C30	1.392 (5)
C9—N6	1.347 (5)	C29—C34	1.397 (5)
C9—H9	0.9500	C30—C31	1.388 (5)
C10—C11	1.384 (5)	C30—H30	0.9500
C10—C15	1.389 (5)	C31—C32	1.376 (6)
C10—P1	1.834 (3)	C31—H31	0.9500
C11—C12	1.383 (5)	C32—C33	1.364 (6)
C11—H11	0.9500	C32—H32	0.9500
C12—C13	1.376 (5)	C33—C34	1.384 (5)
C12—H12	0.9500	C33—H33	0.9500
C13—C14	1.379 (5)	C34—H34	0.9500
C13—H13	0.9500	N1—N2	1.372 (4)
C14—C15	1.386 (5)	N1—Ru1	2.141 (3)
C14—H14	0.9500	N3—N4	1.366 (4)
C15—H15	0.9500	N3—Ru1	2.098 (3)
C16—C21	1.387 (5)	N5—N6	1.357 (4)
C16—C17	1.389 (5)	N5—Ru1	2.134 (3)
C16—P1	1.844 (4)	P1—Ru1	2.3100 (10)
C17—C18	1.384 (5)	Ru1—S2	2.3213 (10)
C17—H17	0.9500	Ru1—S1	2.3962 (10)
			
N2—B1—N4	108.0 (3)	C25—C24—C23	120.6 (4)
N2—B1—N6	107.8 (3)	C25—C24—H24	119.7
N4—B1—N6	108.2 (3)	C23—C24—H24	119.7
N2—B1—H1'	110.9	C26—C25—C24	119.5 (4)
N4—B1—H1'	110.9	C26—C25—H25	120.3
N6—B1—H1'	110.9	C24—C25—H25	120.3
N1—C1—C2	111.5 (3)	C25—C26—C27	120.4 (4)
N1—C1—H1	124.2	C25—C26—H26	119.8
C2—C1—H1	124.2	C27—C26—H26	119.8
C3—C2—C1	104.9 (3)	C26—C27—C22	120.8 (3)
C3—C2—H2	127.5	C26—C27—H27	119.6
C1—C2—H2	127.5	C22—C27—H27	119.6
N2—C3—C2	108.7 (3)	C29—C28—S2	124.0 (3)
N2—C3—H3	125.7	C29—C28—S1	126.4 (3)
C2—C3—H3	125.7	S2—C28—S1	109.6 (2)
N3—C4—C5	110.9 (4)	C30—C29—C34	118.5 (4)
N3—C4—H4	124.5	C30—C29—C28	121.0 (4)
C5—C4—H4	124.5	C34—C29—C28	120.6 (3)
C6—C5—C4	105.1 (3)	C31—C30—C29	120.3 (4)
C6—C5—H5	127.5	C31—C30—H30	119.8
C4—C5—H5	127.5	C29—C30—H30	119.8
N4—C6—C5	108.4 (4)	C32—C31—C30	120.1 (4)
N4—C6—H6	125.8	C32—C31—H31	119.9
C5—C6—H6	125.8	C30—C31—H31	119.9
N5—C7—C8	110.2 (3)	C33—C32—C31	120.3 (4)
N5—C7—H7	124.9	C33—C32—H32	119.9
C8—C7—H7	124.9	C31—C32—H32	119.9
C9—C8—C7	105.3 (3)	C32—C33—C34	120.4 (4)
C9—C8—H8	127.4	C32—C33—H33	119.8
C7—C8—H8	127.4	C34—C33—H33	119.8
N6—C9—C8	108.9 (4)	C33—C34—C29	120.4 (4)
N6—C9—H9	125.6	C33—C34—H34	119.8
C8—C9—H9	125.6	C29—C34—H34	119.8
C11—C10—C15	119.1 (3)	C1—N1—N2	105.1 (3)
C11—C10—P1	123.9 (3)	C1—N1—Ru1	137.7 (2)
C15—C10—P1	116.7 (3)	N2—N1—Ru1	117.1 (2)
C12—C11—C10	120.5 (3)	C3—N2—N1	109.7 (3)
C12—C11—H11	119.7	C3—N2—B1	128.4 (3)
C10—C11—H11	119.7	N1—N2—B1	121.8 (3)
C13—C12—C11	120.2 (4)	C4—N3—N4	106.3 (3)
C13—C12—H12	119.9	C4—N3—Ru1	134.1 (3)
C11—C12—H12	119.9	N4—N3—Ru1	119.3 (2)
C12—C13—C14	119.7 (3)	C6—N4—N3	109.3 (3)
C12—C13—H13	120.1	C6—N4—B1	130.3 (3)
C14—C13—H13	120.1	N3—N4—B1	120.4 (3)
C13—C14—C15	120.4 (4)	C7—N5—N6	106.5 (3)
C13—C14—H14	119.8	C7—N5—Ru1	133.3 (3)
C15—C14—H14	119.8	N6—N5—Ru1	120.1 (2)
C14—C15—C10	120.0 (3)	C9—N6—N5	109.2 (3)
C14—C15—H15	120.0	C9—N6—B1	131.7 (3)
C10—C15—H15	120.0	N5—N6—B1	119.1 (3)
C21—C16—C17	117.3 (3)	C22—P1—C10	104.42 (16)
C21—C16—P1	120.7 (3)	C22—P1—C16	102.15 (16)
C17—C16—P1	122.0 (3)	C10—P1—C16	99.36 (16)
C18—C17—C16	121.4 (4)	C22—P1—Ru1	114.77 (11)
C18—C17—H17	119.3	C10—P1—Ru1	116.19 (12)
C16—C17—H17	119.3	C16—P1—Ru1	117.70 (12)
C19—C18—C17	120.0 (4)	N3—Ru1—N5	85.49 (11)
C19—C18—H18	120.0	N3—Ru1—N1	85.83 (11)
C17—C18—H18	120.0	N5—Ru1—N1	84.61 (11)
C18—C19—C20	120.7 (4)	N3—Ru1—P1	96.44 (8)
C18—C19—H19	119.7	N5—Ru1—P1	177.43 (8)
C20—C19—H19	119.7	N1—Ru1—P1	93.82 (8)
C19—C20—C21	118.9 (4)	N3—Ru1—S2	95.15 (8)
C19—C20—H20	120.5	N5—Ru1—S2	88.31 (8)
C21—C20—H20	120.5	N1—Ru1—S2	172.76 (8)
C16—C21—C20	121.7 (4)	P1—Ru1—S2	93.20 (3)
C16—C21—H21	119.2	N3—Ru1—S1	166.07 (8)
C20—C21—H21	119.2	N5—Ru1—S1	89.71 (8)
C23—C22—C27	118.1 (3)	N1—Ru1—S1	106.76 (8)
C23—C22—P1	122.6 (3)	P1—Ru1—S1	88.79 (3)
C27—C22—P1	118.8 (3)	S2—Ru1—S1	71.61 (3)
C22—C23—C24	120.5 (4)	C28—S1—Ru1	88.02 (12)
C22—C23—H23	119.7	C28—S2—Ru1	90.79 (12)
C24—C23—H23	119.7		
